# Ferrets as a Novel Animal Model for Studying Human Respiratory Syncytial Virus Infections in Immunocompetent and Immunocompromised Hosts

**DOI:** 10.3390/v8060168

**Published:** 2016-06-14

**Authors:** Koert J. Stittelaar, Leon de Waal, Geert van Amerongen, Edwin J.B. Veldhuis Kroeze, Pieter L.A. Fraaij, Carel A. van Baalen, Jeroen J.A. van Kampen, Erhard van der Vries, Albert D.M.E. Osterhaus, Rik L. de Swart

**Affiliations:** 1 Viroclinics Biosciences, 3029 AK Rotterdam, The Netherlands; dewaal@viroclinics.com (L.d.W.); amerongen@viroclinics.com (G.v.A.); veldhuiskroeze@viroclinics.com (E.J.B.V.K.); baalen@viroclinics.com (C.A.v.B.); albert.osterhaus@tiho-hannover.de (A.D.M.E.O.); 2 Department of Viroscience, Erasmus MC, 3015 CN Rotterdam, The Netherlands; p.fraaij@erasmusmc.nl (P.L.A.F.); j.vankampen@erasmusmc.nl (J.J.A.v.K.); erhard.van.der.vries@tiho-hannover.de (E.v.d.V.); r.deswart@erasmusmc.nl (R.L.d.S.); 3 Research Centre for Emerging Infections and Zoonoses, University of Veterinary Medicine, 30559 Hannover, Germany

**Keywords:** respiratory syncytial virus, animal models, ferret, viral loads, immunocompromised host

## Abstract

Human respiratory syncytial virus (HRSV) is an important cause of severe respiratory tract disease in immunocompromised patients. Animal models are indispensable for evaluating novel intervention strategies in this complex patient population. To complement existing models in rodents and non-human primates, we have evaluated the potential benefits of an HRSV infection model in ferrets (*Mustela putorius furo*). Nine- to 12-month-old HRSV-seronegative immunocompetent or immunocompromised ferrets were infected with a low-passage wild-type strain of HRSV subgroup A (10^5^ TCID_50_) administered by intra-tracheal or intra-nasal inoculation. Immune suppression was achieved by bi-daily oral administration of tacrolimus, mycophenolate mofetil, and prednisolone. Throat and nose swabs were collected daily and animals were euthanized four, seven, or 21 days post-infection (DPI). Virus loads were determined by quantitative virus culture and qPCR. We observed efficient HRSV replication in both the upper and lower respiratory tract. In immunocompromised ferrets, virus loads reached higher levels and showed delayed clearance as compared to those in immunocompetent animals. Histopathological evaluation of animals euthanized 4 DPI demonstrated that the virus replicated in the respiratory epithelial cells of the trachea, bronchi, and bronchioles. These animal models can contribute to an assessment of the efficacy and safety of novel HRSV intervention strategies.

## 1. Introduction

Human respiratory syncytial virus (HRSV) is a member of the family *Paramyxoviridae*, genus *Pneumovirus*. Although most HRSV infections remain restricted to the upper respiratory tract (URT) and are associated with relatively mild clinical signs, approximately 0.5%–2% of primary infections in infants below six months of age result in severe bronchiolitis or pneumonia requiring hospitalization [1]. Young infants with a history of premature birth and those with congenital heart or lung disease have an increased risk of developing severe disease upon primary HRSV infection [2]. Another high-risk group of severe HRSV disease is found at the other extreme of age—the frail elderly—with progressively waning immunity, in which HRSV is recognized as an important cause of morbidity and mortality [3]. A third high-risk group of severe HRSV disease is formed of individuals who are immunocompromised, either iatrogenically upon e.g., cell or organ transplantation or cancer treatment, or through inherited or acquired immunodeficiency [4,5,6].

Since the introduction of sensitive and reliable molecular biological diagnostic techniques, a plethora of respiratory viruses has been detected in transplant recipients with clinical symptoms of respiratory tract disease [7]. Immunosuppressive medication given to prevent transplant rejection or graft-*versus*-host disease causes increased susceptibility to infection, and may lead to prolonged virus shedding [8,9,10]. Severity of disease and mortality risk are directly associated with the level and duration of immunodeficiency [11,12]. Lower respiratory tract (LRT) infections with HRSV have especially been associated with high morbidity and mortality in allogenic hematopoietic cell transplant recipients [5,13]. In lung transplant recipients, HRSV infections have been associated with induction of bronchiolitis obliterans syndrome (BOS) [14]. Therapeutic options are currently restricted to administration of ribavirin and/or intravenous immunoglobulin (IVIG) [15,16], although several antiviral compounds are currently in clinical development [2,17].

The population of immunocompromised patients is highly heterogeneous, both in terms of the origin and level of immune suppression and the presence of co-morbidities, which renders studies of the safety and efficacy of novel therapeutics difficult to perform. Preclinical studies in animal models could be instrumental, but all animal models of HRSV infection have limitations [18]. Several animal models have been developed with specific benefits such as high susceptibility (cotton rats, calves, lambs), availability of immunological reagents and knockout strains (mice), and close relationship to humans (non-human primates). However, none of these models fully captures all of these advantages. The human challenge model offers an alternative approach [19,20,21], but for obvious reasons these studies do not allow investigation of severe LRT disease and cannot be performed in individuals belonging to risk groups. Therefore, evaluation of therapeutic safety and efficacy of antiviral compounds in immunocompromised patients largely depends on phase III clinical studies, which are expensive and hard to conduct in this heterogeneous patient group.

We recently developed an animal model for influenza virus infection of immunocompromised patients, by demonstrating that ferrets can be immunocompromised by oral administration of a combination of immunosuppressive compounds used in transplantation patients, including tacrolimus, mycophenolate mofetil (MMF), and prednisolone [8]. Indeed, observations in the immunocompromised ferrets were comparable to those in humans with prolonged influenza virus shedding and emergence of antiviral resistance. The ease of handling the animals allowed for daily collection or respiratory tract swabs, which enabled detailed studies of the kinetics of virus replication. However, whereas ferrets are among the best animal species for modeling human influenza, previous reports of HRSV infections in ferrets have yielded disappointing results. In particular, Prince and Porter infected ferrets of different ages with the Long strain of HRSV, and showed that the virus replicated well in the URT, but poorly in the LRT [22]. Moreover, replication in the LRT proved to be age-dependent and restricted to infant ferrets.

We have infected immunocompetent and immunocompromised ferrets with a low-passage wild-type strain of HRSV subgroup A. We demonstrate that the wild-type virus efficiently replicates in both the URT and LRT of nine- to 12-month-old ferrets. In addition, we demonstrate prolonged virus shedding in immunocompromised animals, as detected both by qPCR and virus isolation.

## 2. Materials and Methods

### 2.1. Ethics Statement and Animal Care and Use

The study protocol and compliance with 3R policies was approved by the Independent Animal Experimentation Ethics Committee in Driebergen, The Netherlands (permit number BXT0014, approval date 8 September 2014). Animals were housed and experiments were conducted in strict compliance with European guidelines (EU directive on animal testing 86/609/EEC) and Dutch legislation (Experiments on Animals Act, 1997). The manuscript was prepared in accordance with the ARRIVE guidelines.

Ferrets (*Mustela putorius furo*) were nine- to 12-month-old purpose-bred females and were seronegative for Aleutian disease virus, circulating influenza virus (sub) types A/H1N1, A/H3N2, and B virus, and RSV subgroup A and B. All ferrets carried a sub-cutaneous chip with their individual animal number. The ferrets, in the weight range of 570–940 g, were maintained in groups of three in cages with a floor area of 12,240 cm^2^ with sawdust bedding (Lignocel^®^, Rettenmaier & Sohne GmbH, Rosenberg, Germany) in accordance with the Code of Practice for the Housing and Care of Animals Bred, Supplied or Used for Scientific Purposes (December 2014).

Animals were provided with cage enrichment (toys and hiding places) and were housed under a 12 h photoperiod regime. Five-week-old female specified pathogen-free cotton rats (*Sigmodon hispidus*) were obtained from Harlan Laboratories, Horst, The Netherlands. Cotton rats were housed individually in filter-top cages with wood fiber bedding, and were provided with paper towels as cage enrichment.

All animals were fed commercial food pellets and offered water *ad libitum*. Animal welfare criteria were verified on a daily basis. Handling of ferrets was performed as follows:

- Twice-daily oral dosing with immunosuppressive medication and daily collection of throat- and nose swabs without sedation;
- Inoculation and preparation for anesthesia: sedation with a mixture of ketamine and medetomidine (IM doses of 0.2 and 0.01 mL/kg body weight (bw), respectively). After inoculation the effect of the medetomidine was antagonized with atipamezole (0.005 mL/kg bw) upon completion of the procedure;
- Blood sampling: sedation with ketamine (intra-muscular [IM] dose of 0.2 mL/kg bw);

Handling of cotton rats was performed under light 4% isoflurane inhalation anesthesia.

### 2.2. Cells and Virus

Human respiratory epithelium carcinoma cells (HEp-2 cells, ATCC^®^ CCL-23™) were cultured in Dulbecco’s Modified Eagle Medium (DMEM; Lonza, Breda, The Netherlands) supplemented with L-glutamine, penicillin, streptomycin and 10% FBS (Greiner Bio-One, Alphen aan Den Rijn, The Netherlands), further referred to as Hep-2 medium.

A wild-type HRSV subgroup A strain was isolated in HEp-2 cells from a nasal lavage of an infant hospitalized at Erasmus MC, Rotterdam, The Netherlands, in 2011. The virus was passaged exclusively in HEp-2 cells. The challenge stock used in the animal experiments was passage 3, and was prepared as follows: monolayers of HEp-2 cells were infected at low multiplicity of infection (MOI, ≤0.01) in serum-free HEp-2 medium for 2 h at 37 °C, after which Hep-2 medium containing 10% FBS was added. After 24 h the serum content was reduced to 2% FBS by removing the medium and adding a serum-free medium. Cytopathic effect (CPE) was followed and cell-free virus was harvested at approximately 50%–75% CPE. Supernatant was clarified by centrifugation for 15 min at 1200 *g*, and subsequently aliquots were snap-frozen using a mixture of 96% ethanol and dry ice and stored at −80 °C. No stabilizers were added to the medium. Virus titers were determined by end-point titration on monolayers of HEp-2 cells and CPE screening six days after infection, and viral titers were calculated using the method of Spearman and Karber. The challenge stock tested negative for a large panel of respiratory human viruses and mycoplasmas by RT-PCR.

### 2.3. Immunosuppressive, Antibiotic, and Antiviral Drugs

The following immunosuppressive drugs were used to suppress the immune system of ferrets: Mycophenolate mofetil (MMF) (CellCept^®^, Roche, Woerden, The Netherlands) powder for infusion, tacrolimus concentrate (5 mg/mL) for infusion (Prograft^®^, Astellas Pharma BV, Leiderdorp, The Netherlands), and prednisolone sodium phosphate (5 mg/mL) oral solution (Hospital Pharmacy, UMCN St Radboud, Nijmegen, The Netherlands). Infusion grade was chosen for MMF and tacrolimus for practical reasons: this provided a liquid formulation of appropriate concentration. To prevent opportunistic bacterial infections due to immune suppression, an antibiotic prophylaxis of amoxicillin supplemented with clavulanic acid (250 mg and 62.5 mg per 5 mL, respectively) oral suspension (Pharmachemie BV, Haarlem, The Netherlands) was used based on dose optimization as described earlier [8]. The HRSV F protein-specific monoclonal antibody Palivizumab (Synagis; AbbVie Ltd., Maidenhead, UK) was reconstituted according to the manufacturer’s instructions.

### 2.4. Human Respiratory Syncytial Virus (HRSV) Infections of Cotton Rats

Cotton rats were inoculated intra-nasally (IN) with 10^5^ TCID_50_ HRSV-A in an inoculum volume of 100 μL, known to result in virus delivery both in the URT and the LRT [23]. Throat swabs were obtained daily, under light 4% isoflurane anesthesia. Necropsies were performed according to a standardized protocol. Nose washes were obtained, followed by lung, nasal turbinate, trachea, and bronchus. Six animals were infected in the framework of this study, and euthanized at 4 (*n* = 3) or 21 (*n* = 3) days post-infection (DPI) by exsanguination under anesthesia.

### 2.5. HRSV Infections of Ferrets: Study Design

Twenty-seven ferrets were randomly assigned to treatment groups of three animals each, divided over two experiments (Table 1). After a one-week acclimatization period, 15 animals were started on the immunosuppression protocol, including daily oral prophylactic antibiotics (10 mg/kg bw amoxicillin and 2.5 mg/kg bw clavulanic acid; starting four days before HRSV infection) and twice-daily oral immunosuppressants (20 mg/kg bw MMF, 0.5 mg/kg bw tacrolimus and 8 mg/kg bw prednisolone; starting three days before HRSV infection), as described in Section 2.3 and reference [8]. One group of immunocompromised animals also received intra-muscular Palivizumab (15 mg/kg bw), on days −2, 0, and 2 relative to HRSV infection. On day 0, all animals were infected with 10^5^ TCID_50_ low-passage wild-type HRSV subgroup A by intra-tracheal (IT) or intra-nasal (IN) inoculation with a volume of 3 or 0.3 mL, respectively. Throat (Copan; rayon tipped) and nose (Copan; polyester tipped) swabs were collected daily in a 3-mL virus transport medium [24], and blood samples were collected −3, −2, 0, 2, 4, 6, 14, and 21 DPI. Animals were euthanized by exsanguination at 4, 7, or 21 DPI. All personnel involved in the collection of study data on a day-to-day basis and all personnel performing the laboratory analysis in which interpretation of the data is required were not aware of the so-called Random Treatment Allocation Key at any time prior to completion of the study. All samples were labeled with a unique sample number.

### 2.6. Samples and Assays

After collection, nose washes, nose swabs, and throat swabs were processed within four hours of sample collection. Infectious virus titers and concentrations of viral RNA were measured by virus isolation and reverse transcription-PCR (RT-PCR), respectively, as previously described [25]. Right lungs and nasal turbinates as well as samples from the trachea and bronchus were weighed and subsequently homogenized with a FastPrep-24 (MP Biomedicals, Eindhoven, The Netherlands) in Hank’s balanced salt solution containing 0.5% lactalbumin, 10% glycerol, 200 U/mL penicillin, 200 µg/mL streptomycin, 100 U/mL polymyxin B sulfate, 250 µg/mL gentamycin, and 50 U/mL nystatin (ICN Pharmaceuticals, Zoetermeer, The Netherlands) and centrifuged briefly before viral load assessment by virus isolation and quantitative PCR (qPCR). Infectious virus titers in tissue are expressed as log10 TCID_50_ per gram tissue, and infectious virus titer in nose washes and swabs are expressed as log10 TCID_50_/mL.

Virus neutralizing (VN) antibody levels were determined using classical end-point neutralization assay, as described previously [26]. Briefly, serial two-fold dilutions of serum samples were incubated with approximately 100 TCID_50_ of RSV (Long strain) for 1 h at 37 °C, HEp-2 cells were added, and the cytopathic effect was monitored during the subsequent seven days. Fifty percent VN titers were calculated from triplicate cultures using the Reed and Muench method.

Formalin-fixed tissue sections were routinely processed, paraffin embedded and sectioned at 3–4 µm, deparaffinized with xylene and rehydrated using graded alcohols, and stained with hematoxylin and eosin for histopathological examination by light microscopy. For immunohistochemistry (IHC) additional serial slides were sectioned simultaneously and incubated for 1 h with a goat anti-HRSV-peroxidase (PO) (Virostat, Portland, ME, USA) polyclonal antibody following antigen retrieval using citric acid buffer. Endogenous PO was blocked with 3% hydrogen peroxide. The bound PO was visualized by incubating slides with 3-amino-9-ethylcarbazole for 10 min as substrate, resulting in a reddish brown granular staining of the cytoplasm of RSV-infected epithelial cells, followed by hematoxylin counterstain. Negative controls were performed in the absence of the antibody.

## 3. Results

### 3.1. HRSV Infection of Immunocompetent Ferrets Results in Productive Virus Replication in the Upper and Lower Respiratory Tract

**Figure 1:**
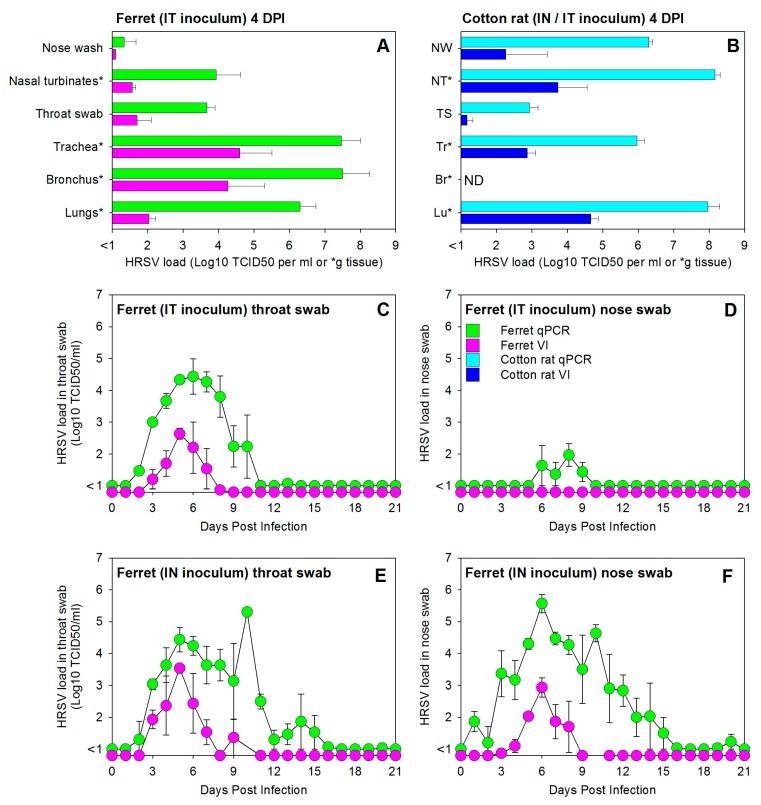
Experimental infection of immunocompetent ferrets with a low-passage wild-type human respiratory syncytial virus (HRSV) subgroup A strain results in productive virus replication in both the upper and lower respiratory tract. (**A**) HRSV loads in the tissues of the upper and lower respiratory tract of immunocompetent ferrets of group #1, infected by intra-tracheal (IT) inoculation and euthanized four days post-infection (DPI); (**B**) HRSV loads in tissues of the upper and lower respiratory tract of immunocompetent cotton rats of group #10, infected with the same virus and dose by IN inoculation in a volume of 100 µL (known to result in both nasal and lung delivery) and euthanized four days post-infection (DPI); HRSV loads in throat swab (**C**,**E**) and nose swab (**D**,**F**) samples collected daily from immunocompetent ferrets infected with a dose of 10^5^ TCID_50_ by IT (**C**,**D**) or IN (**E**,**F**) inoculation (group numbers #2 and #7, respectively). All data are presented as geometric means ± standard error of the mean of groups of three animals (see Table 1). Green bars and symbols (ferrets) and light blue bars (cotton rats) represent HRSV-specific qPCR data, expressed in TCID_50_ equivalents per mL (or per gram tissue for samples labeled with an asterisk in panels **A** and **B**). Purple bars and symbols (ferrets) and dark blue bars (cotton rats) represent HRSV isolation data, expressed in TCID_50_ per mL (or per gram tissue for samples labeled with an asterisk in panels **A** and **B**).

Previous studies suggested that HRSV replicates poorly in the LRT of ferrets [22]. Since these studies were performed by IN inoculation with a laboratory-adapted HRSV strain, we set out to assess whether IT inoculation of adult immunocompetent ferrets with a low-passage wild-type HRSV strain would result in productive virus replication in the LRT. Cotton rats were infected in parallel with the same virus as susceptible controls. In these animals IT infection is difficult to perform, but IN infection using an inoculum size of 100 µL reproducibly results in infection of both URT and LRT [23]. At necropsy 4 DPI, HRSV loads as determined by qPCR or quantitative virus culture from the throat swabs, trachea, and lungs of ferrets (group #1) and cotton rats (group #10) were of the same order of magnitude (Figure 1A–C). Virus loads in nasal swabs and nasal turbinates were substantially higher in cotton rats, which could probably be explained by the differences in routes of inoculation.

An advantage of working with ferrets is the relative ease of handling the animals, which allows for daily collection of throat and nose swabs. In immunocompetent ferrets infected by IT inoculation and euthanized 21 DPI (group #2), HRSV was detected primarily in throat swabs (Figure 1C), although some virus replication was also detected in nose swabs (Figure 1D). In throat swabs HRSV was detectable by isolation until 7 DPI, with peak values at 5 DPI. Using qPCR the virus was detectable until 10 DPI, with peak values at 6 DPI. In nose swabs the virus was exclusively detected by qPCR, and considering the low viral loads this could not be considered as evidence for replication in the URT.

A different pattern was detected in immunocompetent ferrets infected by IN inoculation and euthanized 21 DPI (group #7). In these animals high HRSV loads were detected both by qPCR and virus isolation (Figure 1E,F). Kinetics of the virus isolation curves were largely identical to those detected in IT-inoculated animals, with approximately equal peak values in throat and nasal swabs detected at 5 DPI. Whereas the qPCR curve in throat swabs was almost identical to that of IT infected animals, HRSV loads detected by qPCR in nose swabs reached much higher levels than those in IT inoculated animals, reaching peak values at 6 DPI while remaining detectable until 15 DPI.

### 3.2. HRSV Infection of Immunocompromised Ferrets Results in Higher and Prolonged Virus Replication in the Upper and Lower Respiratory Tract

**Figure 2:**
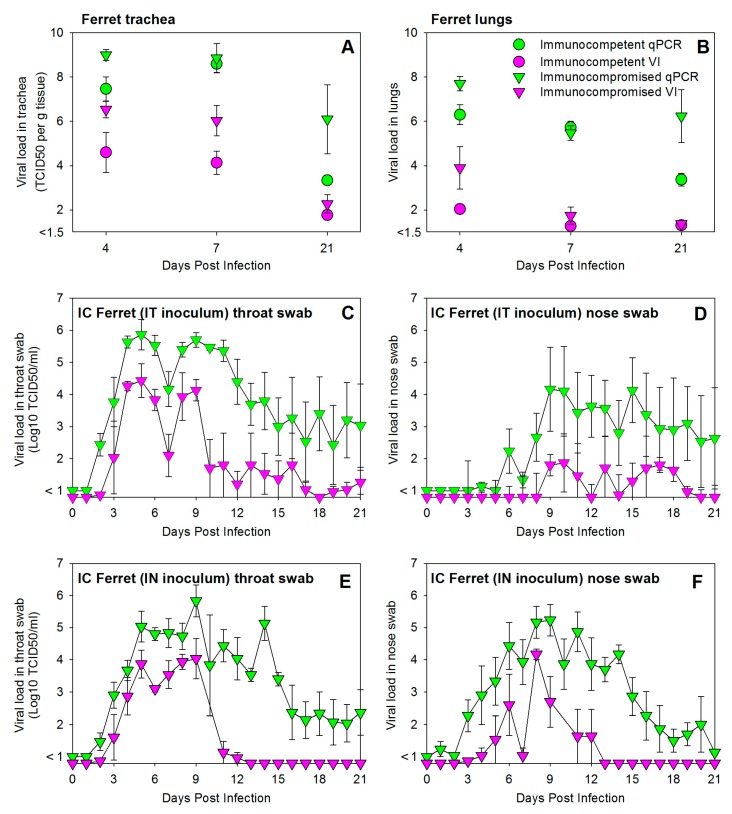
Experimental infection of immunocompromised ferrets with a low-passage wild-type HRSV subgroup A strain results in higher and prolonged virus replication in both the upper and lower respiratory tract. HRSV loads in trachea (**A**) or lungs (**B**) were detected by qPCR and virus isolation after necropsies at 4, 7, or 21 DPI. HRSV loads in throat swab (**C**,**E**) or nose swab (**D**,**F**) samples collected daily from immunocompromised ferrets infected by IT (**C**,**D**) or IN (**E**,**F**) inoculation (group numbers #4 and #9, respectively). All data are presented as geometric means ± standard error of the mean of groups of three animals (Table 1). Immunocompetent and immunocompromised ferrets are shown by circular and triangle-shaped symbols, respectively; qPCR and virus isolation data by green and purple symbols, respectively. Viral loads are shown in TCID_50_ equivalents per gram tissue (**A**,**B**) or TCID_50_ per mL (**C**–**F**).

Comparison of HRSV loads in trachea and lungs of immunocompetent or immunocompromised ferrets infected by IT inoculation and euthanized at 4, 7, or 21 DPI showed higher viral loads in immunocompromised animals, as detected both by quantitative virus isolation and qPCR (Figure 2A,B).

Detection of HRSV in throat and nose swabs of IT-inoculated immunocompromised ferrets euthanized 21 DPI (group #4) showed higher not only but also prolonged presence of viral loads (Figure 2C,D). In contrast to the immunocompetent animals (Figure 1D), virus replication was clearly detected in the URT of these animals (Figure 2D), and apparently resulted in a biphasic viral load curve in throat swabs (Figure 2C). These data suggest that virus detected in throat swabs may have been produced either in the LRT or the URT.

Immunocompromised ferrets infected by IN inoculation and euthanized 21 DPI (group #9) showed a similar pattern of increased and prolonged HRSV replication in throat and nose swabs (Figure 2E,F). Virus loads detected by virus isolation peaked 9 DPI, several days later than those detected in immunocompetent animals (as shown in Figure 1E,F). In both IT- and IN-inoculated animals, virus loads remained detectable by qPCR until 21 DPI in both throat and nose swabs in the majority of animals (2/3 or 3/3 per group).

### 3.3. HRSV Replicates in Respiratory Epithelial Cells of the Ferret

**Figure 3:**
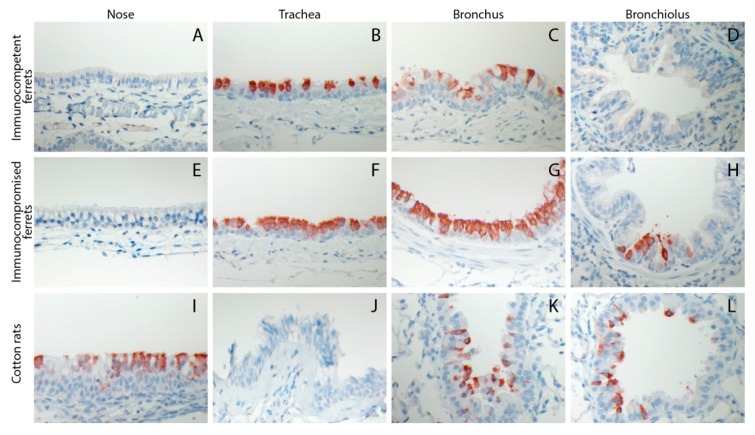
Representative photomicrographs of HRSV antigen expression within the cytoplasm of epithelial cells lining various sections of the respiratory tract of immunocompetent ferrets (**A**–**D**), immunocompromised ferrets (**E**–**H**), or cotton rats (**I**–**L**) euthanized 4 DPI with 10^5^ TCID_50_ of wild-type HRSV by intra-tracheal (ferrets) intra-nasal (cotton rats, large volume inoculum) inoculation. Immunoperoxidase, counterstained with hematoxylin. HRSV antigen is visible as red-brown staining.

Tissue samples collected from ferrets of groups #1 and #3 (infected by IT inoculation and euthanized 4 DPI) and cotton rats of group #10 (infected by IN inoculation and euthanized 4 DPI) were processed for HRSV antigen detection. In immunocompetent ferrets, HRSV-infected ciliated respiratory epithelial cells were detected in the trachea and bronchus, but not in the nose or the bronchioles (Figure 3A–D). In addition, a few neutrophils, few plasmacytes, and few lymphocytes, and sporadic eosinophils were present in the *lamina propria* of the nasal mucosa. Sporadic neutrophils were present in the tracheal epithelial lining and in the bronchiolar and alveolar lumina. In immunocompromised ferrets the same cells were detected in the trachea and bronchus, but at higher levels than those observed in immunocompetent animals, and also in the bronchioles but not in the nose (Figure 3E–H). In addition, sporadic neutrophils were present in the tracheal epithelial lining and in the bronchiolar lumina. In cotton rats HRSV antigen-positive respiratory epithelial cells were detected in the nose, bronchi, and bronchioles, but not in the trachea (Figure 3I–L). In addition, a few neutrophils and mononuclear cells were present in the *lamina propria* of the nasal mucosa and in the alveolar lumina. None of the alveolar epithelial cells of ferrets or cotton rats were found positive for HRSV antigen expression.

### 3.4. Prophylactic Treatment of Immunocompromised Ferrets with Palivizumab Results in Temporary Inhibition of HRSV Replication

**Figure 4:**
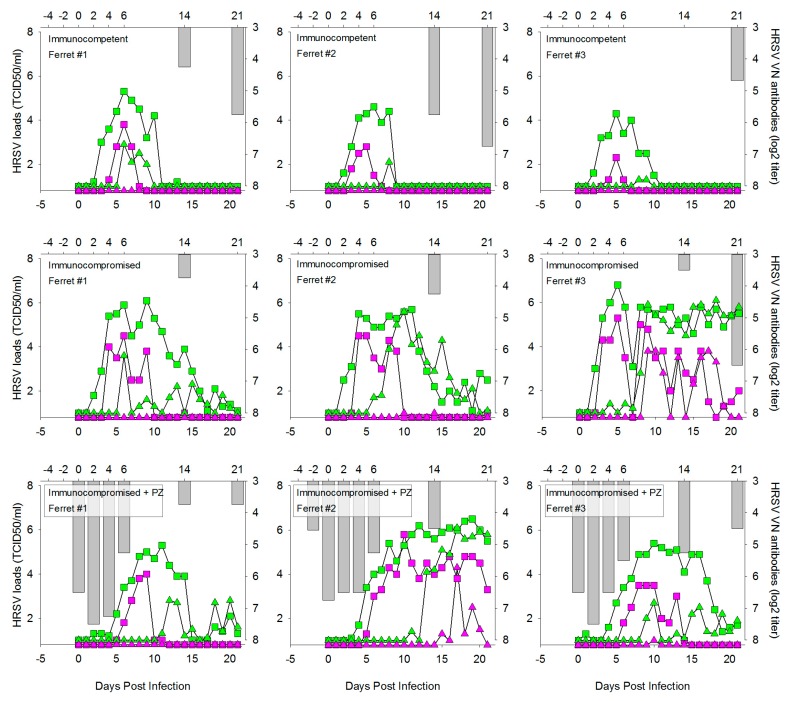
HRSV loads and virus neutralizing (VN) serum antibody responses in individual ferrets at different time points after IT infection with wild-type HRSV subgroup A. HRSV loads (**left** *y*-axis and **bottom** *x*-axis) detected by qPCR and virus isolation are shown as green and purple symbols, respectively. Virus loads in throat and nose swabs are shown by square and triangle symbols, respectively. VN serum antibody levels (**right** *y*-axis and **top** *x*-axis) are shown as reciprocal serum dilution (log2) required for neutralization in 50% of RSV-infected cultures. In groups 2 and 4 VN antibodies are exclusively produced by the animals in response to the HRSV infection, in group 5 they can either be induced by PZ treatment (days -2, 0 and +2) or by *de novo* synthesis by the animals in response to the infection.

Palivizumab (PZ), or Synagis^®^, is an HRSV-specific humanized monoclonal antibody preparation licensed for prophylactic use in infants at high risk of HRSV disease. It is administered monthly by intra-muscular injection, and the first dose should be given prior to the start of the HRSV season. Although PZ does not necessarily prevent infants from becoming infected, it has been shown to help prevent serious lung disease and reduce hospitalization rates [27]. Furthermore, PZ is sometimes used off-label to treat hematopoietic stem cell recipients with HRSV infection. We tested the prophylactic efficacy of PZ in immunocompromised ferrets, by intra-muscular injection −2, 0, and 2 DPI. In Figure 4, viral loads detected by qPCR (green symbols) or virus isolation (purple symbols) in throat swabs (square symbols) or nose swabs (triangle symbols) are plotted over time for IT inoculated animals in group 2 (immunocompetent), group 4 (immunocompromised) and group 5 (immunocompromised and prophylactically treated with PZ). VN antibody titers measured in serum are shown as grey bars. PZ treatment induced significant but short-lasting VN antibody levels in the animals of group 5. As a result, virus loads were initially reduced in these animals.

However, after 5 DPI virus loads started to increase, and a few days later these had reached comparable levels as the animals of group 4. It is interesting to note that, although all immunocompromised animals showed delayed virus clearance as compared to the immunocompetent animals of group 2, we observed substantial individual variation. Both in group 4 and group 5, two out of three animals became virus-negative between two and three weeks after infection, but one out of three animals in each group was unable to clear the infection until after 21 DPI.

## 4. Discussion

In the present study we have demonstrated that ferrets are susceptible to infection with a low-passage wild-type HRSV subgroup A strain. The virus replicated in the epithelial cells of both the upper and lower respiratory tract, which resulted in virus shedding from the upper respiratory tract detectable by quantitative virus culture and quantitative real-time RT-PCR. Furthermore, we have shown that treatment of ferrets with a combination of immunosuppressive medication comparable to the medication used in humans to prevent solid organ transplant rejection resulted in increased and prolonged levels of HRSV replication. The ease of animal handling allowed for daily collection of throat and nose swabs for measurement of viral loads, showing that ferrets may be a useful animal model for the evaluation of novel vaccine strategies as well as for screening the efficacy of novel antiviral compounds in preventing or treating HRSV infection in immunocompetent or immunocompromised patients.

Prince and Porter have previously evaluated ferrets as an animal model of HRSV, by IN inoculation of adult or infant ferrets with the laboratory-adapted Long strain of HRSV [22]. They observed replication in respiratory epithelial cells in the URT of ferrets of all ages, but exclusively detected replication in the LRT in infant ferrets. Interestingly, in these animals HRSV replicated in the alveolar epithelial cells. In IT inoculated ferrets we clearly detected HRSV replication in bronchial epithelial cells, which are also predominant target cells for HRSV in humans [28]. The major differences between the two studies are the route of inoculation and the passage history of the virus. In our study, we have no evidence to show whether IN inoculation of ferrets using an inoculum size of 0.3 mL (150 μL per nostrum) resulted in efficient LRT infection. This will need to be addressed in future experiments in which IN-inoculated animals will be euthanized at early time points. Although HRSV loads were detected in throat swabs of both immunocompetent (Figure 1E) and immunocompromised (Figure 2E) animals, this virus could have derived from either the URT or the LRT. Nevertheless, we expect that the different outcomes of the two studies mainly result from the use of a laboratory-adapted *versus* a wild-type HRSV strain. In other studies, the use of clinical isolates has also been associated with different outcomes of infection both *in vitro* and *in vivo* [29,30,31].

Several animal models have been developed for HRSV, but none of these fully reproduce the pathogenesis of severe HRSV disease observed in humans [32]. This is partly due to the complex pathogenesis in humans, where primary infection can either result in subclinical virus replication, mild disease, or severe disease requiring hospitalization. As we do not fully understand what drives severe disease in humans, it is almost impossible to reproduce this disease in animals. One of the major disadvantages of the ferret model as it is presented here is the apparent lack of clinical signs. It may be possible to induce clinical signs by using a higher virus dose for primary infection, but it is questionable whether that would really mimic human disease. Studies in which clinical signs are of importance can probably better be performed using a natural host model such as the bovine RSV model in calves or lambs [33,34]. A second drawback of the ferret model is the limited availability of immunological reagents, which largely precludes in-depth immunological studies. This is a major restriction, as HRSV bronchiolitis is associated with inflammation and is sometimes considered an immune-mediated disease [35]. Therefore, murine models of HRSV will remain indispensable to support our understanding of the mammalian immune response to HRSV. Finally, our experiments with PZ show that these human immunoglobulins exhibit a short half-life in ferrets, requiring an adjusted dosing schedule to address the efficacy in this animal model. These observations are in accordance with previous studies that found limited effectiveness of human antibodies in the infant ferret model, whereas maternal infection did provide protection against HRSV [36]. Still, the ferret model of HRSV also has a number of strong advantages. First of all, ferrets appear to be highly susceptible to infection with wild-type HRSV. In combination with the ease of handling the animals, which allows for daily collection of throat and nose swabs, this allows sensitive detection of viral loads at high resolution. Importantly, the viral load curves start at undetectable virus levels one or two days after inoculation, demonstrating that the virus detected at later time points is not residual input virus, but truly derives from *de novo* produced HRSV particles. In fact, the ferret studies resemble individual case studies in humans. The fact that the ferrets are outbred non-SPF animals results in a significant level of biological variation, but also brings the model closer to the real situation in humans, in which similar variation is observed. Experiments in ferrets will often include fewer animals than those in rodents, but this is compensated for by the fact that day-to-day collection of clinical specimens is much easier. Finally, when compared to the prototypic highly susceptible animal model for HRSV, the cotton rat, an important difference is the high level of HRSV replication in tracheal epithelial cells, which appears to be absent in cotton rats [23]. Despite their high susceptibility, HRSV-infected cotton rats do not transmit the virus to naive cage mates by direct transmission [37]. We will investigate whether ferrets can be used for transmission studies, which would further strengthen their potential use in preclinical testing of antiviral compounds.

A major strength of the ferret model is the potential to model infection of immunocompromised animals using immunosuppressive medication regularly used in human transplant patients [8]. Respiratory virus infections are described as an important cause of severe respiratory tract disease in immunocompromised patients [5,6,13,38]. The most prevalent respiratory viruses in this population are rhinoviruses and coronaviruses [38], but severe respiratory tract infections are in most cases caused by influenza viruses or paramyxoviruses, including HRSV [39]. With ongoing specialization, academic hospitals have seen a shift in the profile of patients, resulting in an increased number of immunocompromised patients with severe comorbidities. The majority of severe influenza and paramyxovirus infections in adults involves patients with some form of immune dysfunction related to preceding hematopoietic stem cell or solid organ transplantation, or to oncology treatment. The immune suppression results in delayed viral clearance [8,9,40], leading to increased risk of lower respiratory tract infection, potential for development of antiviral resistance, escape from neutralizing antibodies, and risk of nosocomial spread [41,42]. Our findings obtained using the immunocompromised ferrets resemble the increased viral loads and delayed viral clearance in humans, as demonstrated by the day-to-day sampling of the immunocompromised ferrets. Animal models for HRSV infection of the immunocompromised host have also been developed in mice and cotton rats, resulting in more severe lung lesions or prolonged RSV replication, respectively [43,44]. However, these models were based on the use of cyclophosphamide monotherapy, which is less commonly used in humans than combination immune suppressive therapies. As ferrets provide an important animal model for human influenza, we previously developed a protocol to induce immune suppression in ferrets using medication routinely used in human transplant patients, and demonstrated prolonged influenza virus replication [8]. Moreover, we also demonstrated that the use of antiviral compounds could result in development of drug resistance. Based on the HRSV data presented in the current manuscript, we believe that the HRSV model in ferrets can be used in parallel with the influenza model for preclinical studies of antiviral efficacy and development of drug resistance.

## 5. Conclusions

In conclusion, the HRSV model in immunocompetent and immunocompromised ferrets can complement existing animal models and contribute to the efficacy and safety of novel HRSV intervention strategies.

## Figures and Tables

**Table 1:** Study design.

**Exp #**	**Group #**	**# Ferrets**	**Route**	**Dose**	**IC**	**PZ**	**Necropsy**
1	1	3	IT	10^5^ TCID_50_	No	No	4 DPI
1	2	3	IT	10^5^ TCID_50_	No	No	21 DPI
1	3	3	IT	10^5^ TCID_50_	Yes	No	4 DPI
1	4	3	IT	10^5^ TCID_50_	Yes	No	21 DPI
1	5	3	IT	10^5^ TCID_50_	Yes	Yes	21 DPI
2	6	3	IT	10^5^ TCID_50_	No	No	7 DPI
2	7	3	IN	10^5^ TCID_50_	No	No	21 DPI
2	8	3	IT	10^5^ TCID_50_	Yes	No	7 DPI
2	9	3	IN	10^5^ TCID_50_	Yes	No	21 DPI
**Exp #**	**Group #**	**# Cotton rats**	**Route**	**Dose**	**IC**	**PZ**	**Necropsy**
1	10	3	IN *	10^5^ TCID_50_	No	No	4 DPI
1	11	3	IN *	10^5^ TCID_50_	No	No	21 DPI

DPI = days post-infection; Exp # = experiment number; IT = intra-tracheal; IN = intra-nasal; IC = immunocompromised; PZ = Palivizumab; * IN infection of cotton rats with an inoculum volume of 100 µL results in human respiratory syncytial virus (HRSV) delivery to both the upper respiratory tract (URT) and lower respiratory tract (LRT).
